# Valley‐scale hydrogeomorphology drives river fish assemblage variation in Mongolia

**DOI:** 10.1002/ece3.7505

**Published:** 2021-03-30

**Authors:** Alain Maasri, Mark Pyron, Emily R. Arsenault, James H. Thorp, Bud Mendsaikhan, Flavia Tromboni, Mario Minder, Scott J. Kenner, John Costello, Sudeep Chandra, Amarbat Otgonganbat, Bazartseren Boldgiv

**Affiliations:** ^1^ Department of Ecosystem Research Leibniz Institute of Freshwater Ecology and Inland Fisheries (IGB) Berlin Germany; ^2^ The Academy of Natural Sciences of Drexel University Philadelphia PA USA; ^3^ Department of Biology Ball State University Muncie IN USA; ^4^ Kansas Biological Survey and the Department of Ecology and Evolutionary Biology University of Kansas Lawrence KS USA; ^5^ Institute of Geography and Geoecology Mongolian Academy of Sciences Ulaanbaatar Mongolia; ^6^ Global Water Center and Department of Biology University of Nevada Reno NV USA; ^7^ Department of Civil and Environmental Engineering South Dakota School of Mines & Technology Rapid City SD USA; ^8^ Department of Biology National University of Mongolia Ulaanbaatar Mongolia

**Keywords:** beta diversity, ecoregional differences, functional process zones, fish biodiversity, macrosystem ecology, riverine ecosystem synthesis

## Abstract

River hydrogeomorphology is a major driver shaping biodiversity and community composition. Here, we examine how hydrogeomorphic heterogeneity expressed by Functional Process Zones (FPZs) in river networks is associated with fish assemblage variation. We examined this association in two distinct ecoregions in Mongolia expected to display different gradients of river network hydrogeomorphic heterogeneity. We delineated FPZs by extracting valley‐scale hydrogeomorphic variables at 10 km sample intervals in forest steppe (FS) and in grassland (G) river networks. We sampled fish assemblages and examined variation associated with changes in gradients of hydrogeomorphology as expressed by the FPZs. Thus, we examined assemblage variation as patterns of occurrence‐ and abundance‐based beta diversities for the taxonomic composition of assemblages and as functional beta diversity. Overall, we delineated 5 and 6 FPZs in river networks of the FS and G, respectively. Eight fish species were found in the FS river network and seventeen in the G, four of them common to both ecoregions. Functional richness was correspondingly higher in the G river network. Variation in the taxonomic composition of assemblages was driven by species turnover and was only significant in the G river network. Abundance‐based taxonomic variation was significant in river networks of both ecoregions, while the functional beta diversity results were inconclusive. We show that valley‐scale hydrogeomorphology is a significant driver of variation in fish assemblages at a macrosystem scale. Both changes in the composition of fish assemblages and the carrying capacity of the river network were driven by valley‐scale hydrogeomorphic variables. River network hydrogeomorphology as accounted for in the study has, therefore, the potential to inform macrosystem scale community ecology research and conservation efforts.

## Graphical abstract

Valley‐scale hydrogeomorphology plays a fundamental role structuring river fish assemblages. These pictures show two distinct Functional Process Zones of the hydrogeomorphic gradient established in the Delgermörön river, a major tributary of the Selenge river network. Picture credit: Emily Arsenault, Mongolia, 2017.

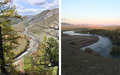

## 1 INTRODUCTION

Fish assemblages differ among ecoregions due to diverse evolutionary origins and historical dispersal routes (Wiley & Mayden, 1985). In addition, stream fish assemblages are the product of selection along environmental gradients driven by differences in biological, chemical, topographic, and in‐stream physical variables that occur at multiple scales associated with landscape, valley, and local stream conditions (Fausch et al., 2002; Frissell et al., 1986; Gido et al., 2006; Gorman & Karr, 1978; Sullivan et al., 2006). Most of these in‐stream physical variables reflect the attributes of fluvial hydrogeomorphology resulting from valley‐scale variables that include floodplain structure and valley slope and shape (Baxter & Hauer, 2000; Boys & Thoms, 2006). The effects of environmental gradients on fish assemblages are well‐studied along longitudinal river gradients (Jackson et al., 2001). At intermediate and small spatial scales, local environmental gradients (e.g., habitat and microhabitat use) filter species from a regional pool leading to a realized assemblage at a local scale (Poff, 1997). Such gradients, while being more temporally variable, are often easier to measure and to correlate with fish assemblage than large‐scale processes (Lamouroux et al., 1999). Among large‐scale processes, the longitudinal succession of fish species (i.e., species turnover) has been widely examined and validated. In general, longitudinal changes (or zonation) in fish assemblages are thought to be predictable as responses to longitudinal continuous gradients in water temperature, channel morphology, and water velocity. In contrast, distinct changes in fish assemblages at smaller spatial extents are considered to be responses to abrupt discontinuities in stream hydrogeomorphology (e.g., Belliard et al., 1997; Torgersen et al., 2006; Zbinden & Matthews, 2017). Such discontinuities, primarily driven by valley‐scale hydrogeomorphic features, often better describe stream biocomplexity of dynamic interactions between communities and environmental physical parameters.

The Riverine Ecosystem Synthesis (RES, Thorp et al., 2008) portrays streams as a repeatable, and only partially predictable, succession of large hydrogeomorphic patches formed by drivers including regional geology, valley conditions, channel and valley geomorphic structures, and climatic and hydrologic patterns (Thoms et al., 2018; Thorp et al., 2006, 2008). The first tenet of the RES predicts that species distribution in a river network is associated primarily with the distribution of large spatial patches formed principally by hydrogeomorphic forces and modified by climate and vegetation. These hydrogeomorphic patches, often called Functional Process Zones (FPZs), revealed significant associations with discrete stream assemblages of fishes (Elgueta et al., 2019) and macroinvertebrates (Maasri et al., 2019, 2021). However, how the heterogeneity of FPZs affects the organization of fish assemblages at the scale of a river macrosystem has not been adequately explored yet.

Here, we examine how hydrogeomorphic heterogeneity, expressed by FPZs in river networks, is associated with fish assemblage variation. We examined this relationship in two distinct ecoregions in Mongolia: forest steppe (FS) and grassland (G). Mongolian rivers represent ideal study sites to investigate how river network hydrogeomorphology drives changes in fish assemblages since most Mongolian rivers are relatively unimpacted by human activities. Mongolian rivers constitute an opportunity to study river systems prior to any development largely because of sparse human population density and extreme remoteness (Mercado‐Silva et al., 2008). Moreover, these rivers generally lack impoundments and introduced species. The available literature on fish assemblages in Mongolian rivers is scarce, with fish assemblages only being explored in a few publications, including identification and occurrences (Kottelat, 2006; Mendsaikhan et al., 2017), ecology (Kaus et al., 2018; Mendsaikhan et al., 2016; Mercado‐Silva et al., 2008; Olson et al., 2016), conservation (Jensen et al., 2009), and genetics (Dulmaa et al., 2016; Kaus et al., 2019; Roman et al., 2018). However, variation of fish assemblages at the scale of entire river networks is largely unknown for most Mongolian drainages. Moreover, the variation of fish assemblages with hydrogeomorphology is still vastly unexplored.

**FIGURE 1:**
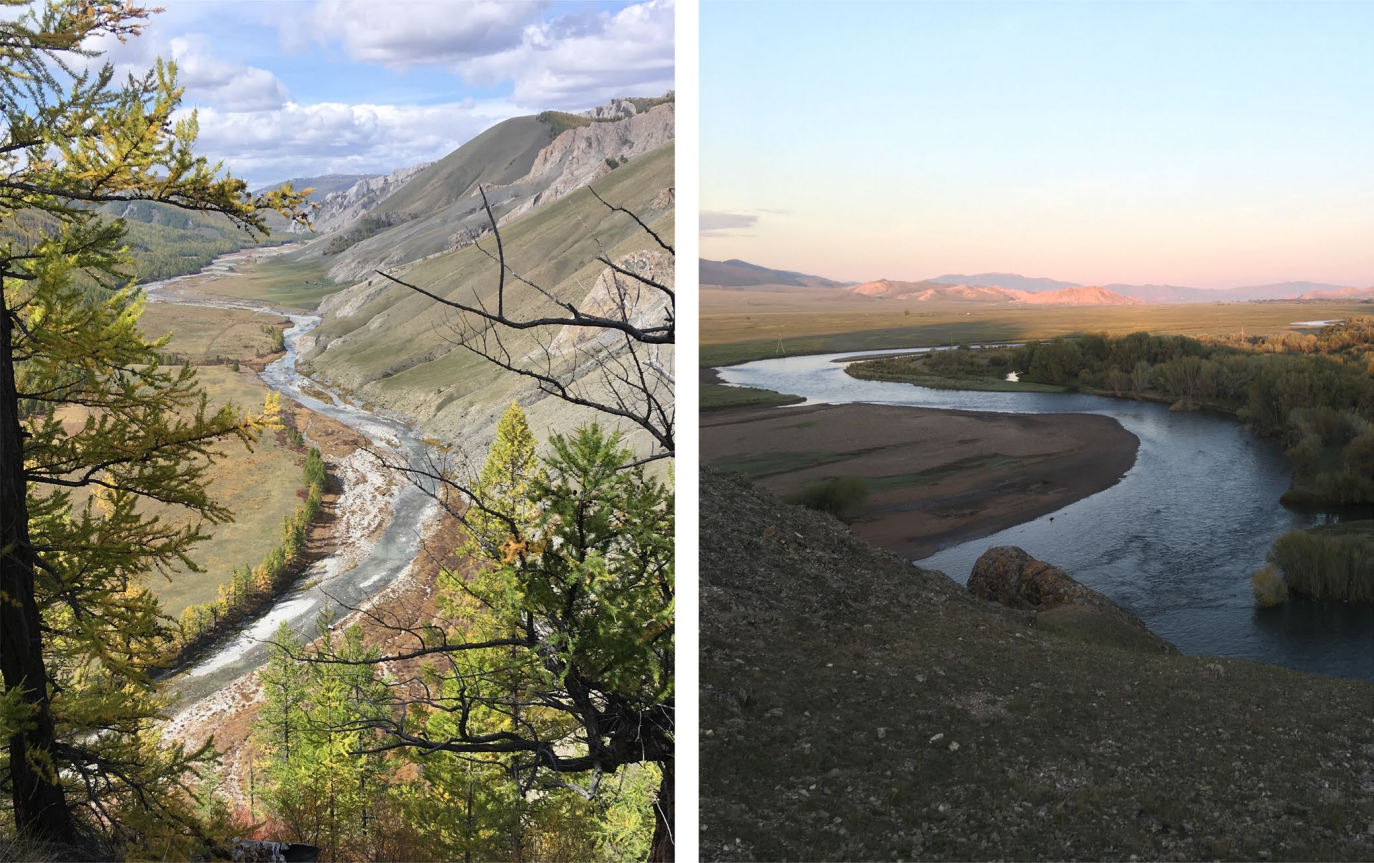
Picture of two distinct Functional Process Zones of the hydrogeomorphic gradient established in the Delgermörön river, a major tributary of the Selenge river network. Picture credit: Emily Arsenault, Mongolia, 2017

We predict that variation in hydrogeomorphology among river networks located in two different ecoregions of Mongolia will have resulted in unique associations between fish habitats and fish assemblages. For example, the river network of the FS ecoregion tends to be high gradient, with narrow valleys that result in constrained banks, and displays higher seasonal hydrological variation (Figure 1). Fish habitats in this river network should consist of large substrata, high current velocity, and distinct riffle‐run units. In contrast, the river network of the G ecoregion should be low gradient and characterized by wide valleys with unconstrained banks, and more predictable hydrological variation. Fish habitats in the G river network should consist of small substrata, low current velocity, side channels, and less distinct riffle‐run units. Thus, we hypothesized that: (a) Fish assemblages of both ecoregions vary with river hydrogeomorphology at the valley scale when the variation of assemblages is evaluated as patterns of beta diversity, and (b) such variation should result from discrete changes in both taxonomic and functional compositions of fish assemblages. Given that the range of valley‐scale hydrogeomorphic variables is expected to be different among ecoregions, we hypothesized that (c) the variation of fish assemblages in association with the hydrogeomorphic gradients displayed by river networks would be different in the two ecoregions. Thus, greater range of valley‐scale hydrogeomorphic heterogeneity should result in a greater variation of fish assemblages in the river network .

## 2 METHODS

### 2.1 River and site selection

**FIGURE 2:**
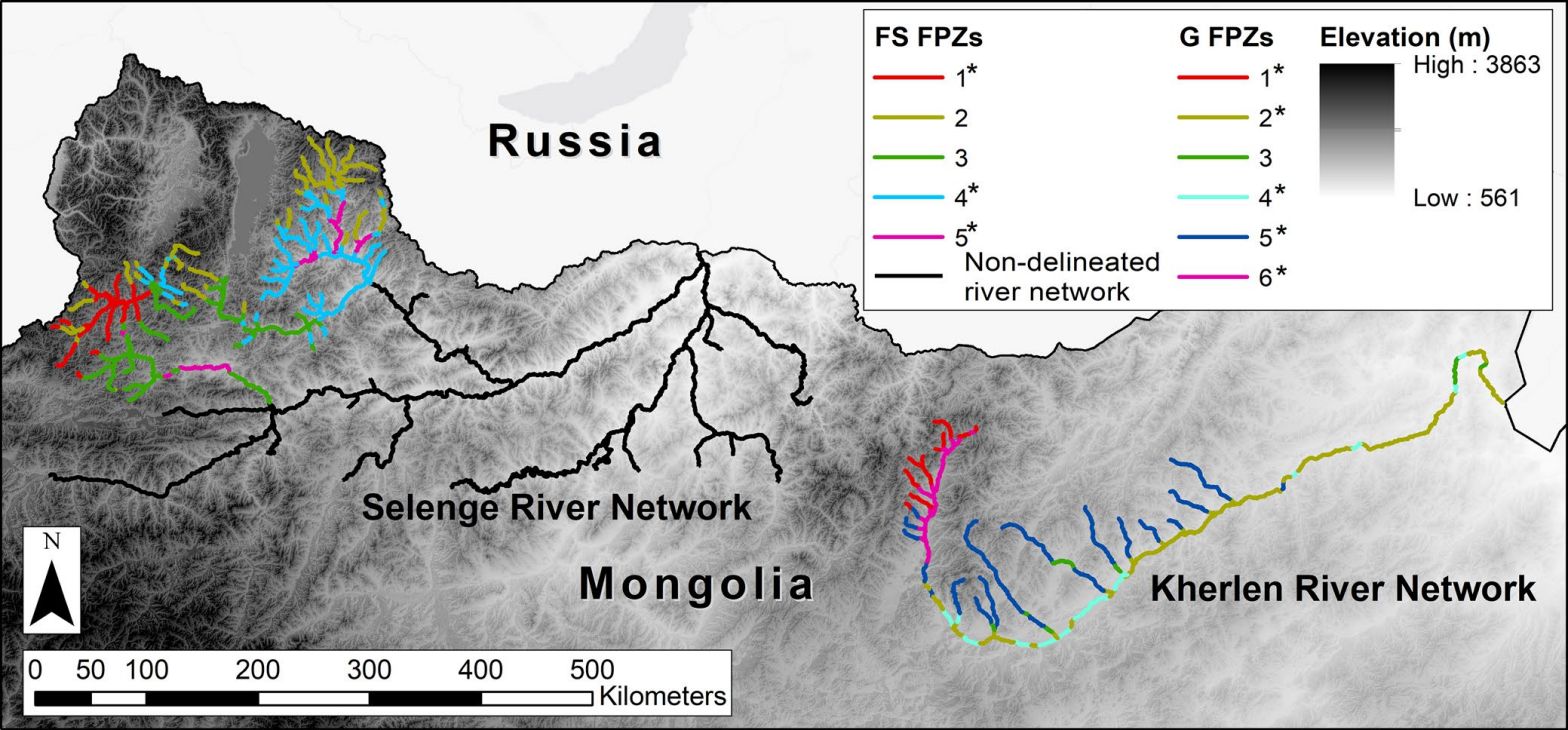
Map showing the FPZ delineation, from left to right, in the forest steppe river network (FS; portion of the Selenge river network) and the grassland river network (G; the Kherlen river network) in Mongolia. Sampled FPZs for fish assemblages are marked with an asterisk (*), and the color code used in the figure for the different FPZs is the same used in Appendix S2. Elevation (a.s.l. in m) is represented as a grayscale shade

We examined fish assemblages in river networks of two ecoregions of the temperate steppe biome in Mongolia. We sampled the Kherlen River network flowing across the grassland ecoregion (G) of eastern Mongolia and the Delgermörön and Eg Rivers, major tributaries of the Selenge River network, flowing across the forest steppe ecoregion (FS) of northern Mongolia (Figure 2). The Kherlen River network flows east into China and empties into Lake Hulum (called also Dalai Nuur), while the Selenge River network flows north into Russia and empties into Lake Baikal. These two major river networks are considered representative of rivers of the two ecoregions and are expected to display different fish assemblages.

We delineated Functional Process Zones (FPZs, also called hydrogeomorphic patches; Thorp et al., 2006; Thorp et al., 2008) using the GIS‐based program RESonate (Williams et al., 2013) to extract valley‐scale hydrogeomorphic and environmental variables from existing geospatial datasets. Primary geospatial data inputs to the RESonate included the following: mean annual precipitation extracted from WorldClim (30 arc‐second), digital elevation model (DEM, Shuttle Radar Topography Mission (SRTM, 30‐m resolution)), geological maps (The National Snow and Ice Data Center's Northern circumpolar soils map), and streamline data (modeled from SRTM DEMs). For the delineation of FPZs, we used the ten most influential variables for valley‐scale hydrogeomorphology. We extracted these variables at 10‐km sample intervals: elevation, mean annual precipitation, geology (i.e., soil orders and geological characteristics), valley width, valley floor width (i.e., floodplain), valley width‐to‐valley floor width ratio, river channel sinuosity, right valley slope, left valley slope, and down valley slope. For each river network, data were normalized to a 0–1 scale and a dissimilarity matrix was generated using a Gower dissimilarity transformation (Gower, 1971). The Gower transformation is recommended for nonbiological data when the measures are range‐standardized (Thoms & Parsons, 2003). The dissimilarity matrix was used in a hierarchical clustering following the Ward linkage method, as it provided the best partitioning of cluster groups (Murtagh & Legendre, 2014). Additionally, we used a principal component analysis (PCA) to identify the contributive variables most important for group partitioning and to describe the cluster groups based on the ten variables identified above. Groups were later mapped to allow for identification of sampling sites (see below). We performed the clustering of FPZ groups using the *cluster* package (version 2.1.0) (Maechler et al., 2021) and the PCA using the *FactoMineR* package (version 1.42) (Lê et al., 2008) in R version 3.6.3 (R Core Team, 2020). We mapped the resulting groups using ArcGIS (version 10.5).

To test our hypotheses, we established hydrogeomorphic distances among the different FPZs occurring in the FS and G river networks. We used PCAs computed on the valley‐scale hydrogeomorphic variables to establish hydrogeomorphic distances as pairwise Euclidean distances between FPZs in the PCA bi‐dimensional plot. Pairwise Euclidean distances were established separately among FPZs in each ecoregion and were measured as distances between centroids summarizing the distribution of river sections in the bi‐dimensional plot. Euclidean distances were later used to examine the linear regression models between hydrogeomorphic distances and beta diversity (β‐diversity) metrics.

### 2.2 Fish collections and traits

We collected fishes from reaches representing three FPZs in the FS river network and five FPZs in the G river network (see below). Sampling FPZs was constrained by accessibility to stream sites in a remote region and by time limitation during a one‐month field expedition to sample each ecoregion. We established reaches for fish collections measuring at least 20 times the mean wetted width of the river at the sampling area. Selected reaches were representative of FPZs based on a visual inspection. We collected fishes by single‐pass backpack electrofishing supplemented with angling (Ball State University IACUC #126193) following the American Fisheries Society standard collection protocols (Bonar et al., 2009) and reported fish abundance as fish‐per‐meter sampled. We sampled fishes during one‐month expeditions in each of the river networks, during the month of August in 2017 for the FS and 2019 for the G.

Fish species identifications and ecological and biological traits were determined according to Mendsaikhan et al., (2017), and reproductive traits were referenced from Balon (1975). Continuous fish traits of longevity, fecundity, and maximal length were transformed to categorical traits as described in Appendix S1. In total, we used 10 traits described with 41 modalities for subsequent trait analyses.

### 2.3 Community data analyses

We calculated the taxonomic and functional alpha diversities for the sampled FPZs in each ecoregion. Taxonomic alpha diversity (taxonomic richness, TRic) is expressed as the total number of fish species occurring in an FPZ, while functional alpha diversity was examined using the computed component of functional richness (FRic). FRic is a multidimensional measure that represents the amount of functional trait space filled by a community. The functional trait space is therefore computed based on a convex hull volume algorithm as described by Villéger et al. (2008).

We examined variation in fish assemblages, as patterns of β‐diversity, along the hydrogeomorphic gradients displayed by the river networks in each ecoregion. We computed (a) taxonomic β‐diversity metrics based on species occurrence, (b) taxonomic quantitative β‐diversity metrics based on species abundance, and (c) functional β‐diversity metrics accounting for the different ecological and biological traits expressed by the fish assemblages. We expected that taxonomic β‐diversity indices for occurrence data and on abundance data would provide complementary information reflecting not only how replacement and richness difference occur among assemblages but also how the carrying capacities of FPZs change. Quantitative and qualitative pairwise taxonomic β‐diversity metrics (for both occurrence data and abundance data) were computed as proposed by Baselga (2010) and Baselga (2013) in order to examine assemblage compositional differences between FPZs. Thus, on occurrence data we computed (a) the Sørensen dissimilarity index (*β*_sor_) to account for total compositional variation between assemblages of two FPZs, (b) the Simpson dissimilarity index (*β*_sim_) to capture only compositional changes due to taxa turnover, and (c) the nested‐resultant dissimilarity (*β*_sne_) calculated as the difference between *β*_sor_ and *β*_sim_. On abundance data, we computed (d) the Ružička dissimilarity index (*β*_ruz_) to capture total abundance variation between FPZs in terms of their composition, (e) the balanced change in species abundance (*β*_ruz‐bal_), and (f) the unidirectional abundance gradient (*β*_ruz‐gra_). *β*_ruz_ results from the summation of *β*_ruz‐bal_ and *β*_ruz‐gra_, two antithetic sources of dissimilarity. We applied Hellinger transformation on abundance data before computing β‐diversity indices. Hellinger transformation offers a better compromise between linearity and resolution and has been recommended when simulated across ecological and geographic gradients (Legendre & Gallagher, 2001).

Pairwise functional β‐diversity consisted of examining dissimilarities between assemblages of FPZs based on variation in volumes of the intersections of convex hulls in a multidimensional functional trait space as described by Villéger et al. (2013). For each ecoregion, we calculated a species‐by‐species Euclidean distance matrix on the trait data. Then, we used a metric multidimensional scaling analysis (MDS, which is equivalent to a principal coordinates analysis), to break correlations between traits creating, therefore, orthogonal synthetic traits (MDS axes) that represented spectra of fish ecological and biological niches. Coordinates of species along the first two MDS axes were used later on as inputs in the functional dissimilarity equations (Villéger et al., 2013).

We performed all computations in R version 3.6.3 (R Core Team, 2020) and calculated the FRic using the *FD* package (version 1.0.12) (Laliberté et al., 2014), the β‐diversity metrics using the *betapart* package (version 1.5.1) (Baselga & Orme, 2012), and the MDS using the *vegan* package (version 2.5‐6) (Oksanen et al., 2019).

## 3 RESULTS

The FPZ delineation of the forest steppe (FS) and grassland (G) river networks spanned 3,380 and 2,390 km of linear river network distances, respectively. FS river sections were mainly characterized by higher elevation and valley slopes, while G river sections were characterized by wide valleys and floodplains (i.e., valley floor, see Appendix S2‐1). Using the hierarchical method, we established five and six FPZs for the FS and G river networks, respectively. The most contributing variable for FPZ delineation in FS was valley width, followed by elevation and valley floor width (Appendix S2‐2). For FPZ delineation in G, valley floor width was the most contributing variable, followed by valley width and elevation.

**FIGURE 3:**
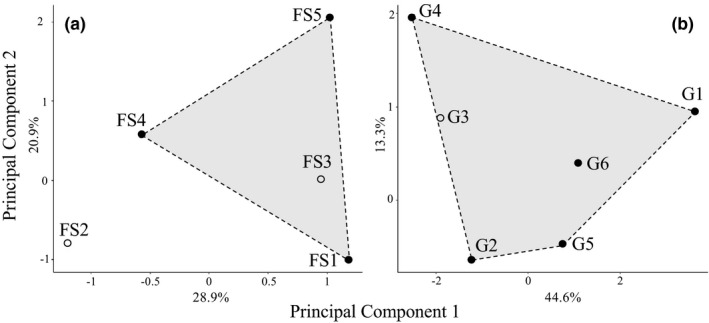
Scatter plots showing the distribution of Functional Process Zones (FPZs) in forest steppe (plot A, FPZs: FS1–5) and grassland (plot B, FPZs: G 1–6) rivers. Black dots indicate FPZs sampled for fish communities and included in the analysis. Polygons are representations of the hydrogeomorphic gradients (see Methods section) covered in the analysis

Euclidean distances, representing hydrogeomorphic distances, among FPZ were established in a PCA bi‐plots summarizing 49.8%, and 57.9% of the total variance for FS and G, respectively (Figure 3). The convex hulls delimiting the sampled FPZs, a representation of the portion of hydrogeomorphic gradients accounted for in our analysis, show that the entire hydrogeomorphic gradient expressed by FPZs in the G river network was accounted for because all FPZs fall within the total convex hull area. In contrast, we were able to account for only a portion of the hydrogeomorphic gradient expressed by FPZs in the FS river network.

**FIGURE 4:**
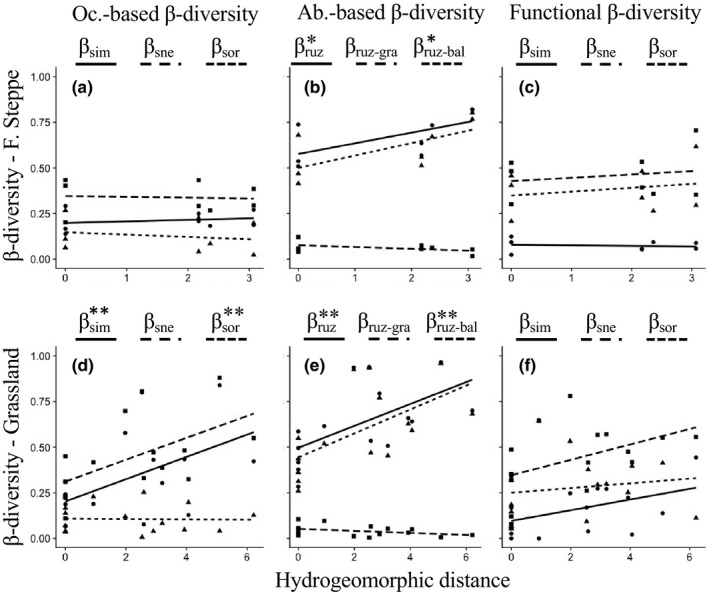
Plots showing linear regressions between the hydrogeomorphic distances established between FPZs in forest steppe (plots a, b, c) and grassland river networks (plots d, e, f) and β‐diversity metrics. Pairwise Euclidean distances representing hydrogeomorphic distances are plotted on the x‐axes and the β‐diversity metrics on the y‐axes. Plots are organized into three columns, showing regressions for the occurrence‐based β‐diversity (plot a and d), the abundance‐based β‐diversity (plot b and e), and the functional β‐diversity (plot c and f). The β‐diversity metrics are introduced in the Methods section and are shown here with different line patterns. Asterisks (*) next to the metrics signal statistical significance of the linear regression, with one and two asterisks indicating significance at 10% and 5%, respectively

We sampled fish assemblages from three and five FPZs from the G and FS river networks, respectively (Appendix S2‐3). We collected a total of 21 fish species in the two sampled river networks (Appendix S1), of which only four species were shared by both ecoregions. Eight species were found in the FS and seventeen species in G. Overall, higher TRic values were found in FPZs of G compared to FS. On average, 3.9 (±0.5) species were found per FPZ in the FS river network and 6.1 (±1.6) species per FPZ in the G river network. Taxonomic variation as expressed by the occurrence‐based β‐diversity metrics shows increasing dissimilarity in species composition across the hydrogeomorphic gradient displayed by FPZs in the G river network (Figure 4d). This increase in dissimilarity (*β_s_*_or_: *R* ^2^ = 0.27, *p* = 0.02) was driven only by species turnover (*β*_sim_: *R* ^2^ = 0.20, *p* = 0.05). No dissimilarity was observed across the hydrogeomorphic gradient displayed by FPZs in the FS river network (Figure 4a). Abundance‐based β‐diversity metrics show increasing dissimilarity in total abundance variation for both G (*β*_ruz_: *R* ^2^ = 0.30, *p* = 0.02) and FS (*β*_ruz_: *R* ^2^ = 0.32, *p* = 0.08) river networks. This change is attributed to increasing dissimilarity in the balanced change in species abundance (*β*_ruz‐bal_: *R* ^2^ = 0.34, *p* = 0.07, and *R* ^2^ = 0.31, *p* = 0.01 for FS and G river networks, respectively).

Overall, higher FRic values were found in FPZs of G compared to FS, with FRic values of 15.3 (±11.3) and 9.5 (±5.7), respectively. The results of functional β‐diversity were, however, inconclusive (Figure 4c and f). No significant linear regressions were established between any of the β‐diversity metrics and the hydrogeomorphic gradients displayed by FPZs in FS and G river networks.

## 4 DISCUSSION

Our study demonstrated that hydrogeomorphic gradients, expressed by the range of hydrogeomorphic characteristics of Functional Process Zones (FPZs) in river networks, are drivers of variation in fish assemblages. These gradients of discrete hydrogeomorphic patches established at the valley scale should not be confused with the longitudinal river zonation as expressed by the River Continuum Concept (RCC, Vannote et al., 1980), where longitudinal changes in fish assemblages along the river continuum have already been validated (Goldstein & Meador, 2004; Oberdorff et al., 1993). The RCC has predicted changes in communities driven by longitudinal changes in carbon and food sources, and parameters associated with changes in elevation (e.g., dissolved oxygen, temperature, and flow velocity). However, it failed to underline the role of hydrogeomorphology in driving changes in communities. Here, we demonstrate how valley‐scale hydrogeomorphology, not necessarily associated with longitudinal changes reflected by a river continuum, drives change in fish communities. Thus, the observed changes in β‐diversity patterns associated with hydrogeomorphic gradients in river networks of forest steppe (FS) and grassland (G) ecoregions support our first hypothesis.

Mongolian fish assemblages of FS and G river networks are expected to have low species richness compared to watersheds at lower latitudes (Willig et al., 2003). We collected 21 out of 33 fish species known to occur in these two ecoregions (Mendsaikhan et al., 2017). The twelve species we did not collect are large‐bodied or rare fishes and not likely to be collected easily with our fishing methods (Appendix S1).

We found discrete fish assemblages associated with FPZs as previously reported by Elgueta et al. (2019). However, variation in the occurrence of fish species across the hydrogeomorphic gradient was only visible in the G river network and was mainly driven by species turnover (i.e., species replacement). Such contribution of species turnover to assemblage variation has been previously observed across different ecoregions and was associated mainly with elevation changes (Barboza & Villalobos, 2018; Herrera‐Pérez et al., 2019). No occurrence‐based taxonomic variation was associated with the hydrogeomorphic gradient established for the FS river network. This difference can be partially explained by differences in the delineation of river networks between the two ecoregions. The entire river network (i.e., from headwaters to near mouth) was delineated in G. Comparably, only a portion of the river network in FS was delineated because of the very large size of this river network and for practical sampling constraints in the anticipated month‐long expedition in Mongolia. Yet, the delineated portion of the FS river network is still larger than the entire river network in G (see results, 3,380 and 2,390 km for FS and G, respectively). We can consider that this delineation characterized mainly the upper portion of the Selenge River network, a major river network in Mongolia that drains an estimate of 60% of the inflow to Lake Baikal in neighboring Russia. Accordingly, we believe that the hydrogeomorphic gradient established for FS displays only partially the gradient of available FPZs, and the fish species collected in FS similarly characterize only the upper part of this river network.

In contrast, variation in abundance‐based β‐diversity metrics was associated with the hydrogeomorphic gradients established in both ecoregions. The abundance‐based variation shows changes in the balance of abundances among species where the individuals of some species in one FPZ are substituted by the same number of individuals of different species in another FPZ (see, Baselga, 2013). Such variation can be interpreted as changes in the carrying capacity of FPZs, a direct feature of habitat characteristics and availability (Cramer & Ackerman, 2009). The availability and diversity of suitable habitats will typically determine the carrying capacity of FPZs for fishes, and we predicted that habitat characteristics would be directly linked to valley‐scale hydrogeomorphology as accounted for in this study. Exceptions can certainly arise when river connectivity is an issue; however, this is unlikely among the Mongolian drainages we examined (Kaus et al., 2018; Maasri et al., 2018). We can conclude that variation in the taxonomic composition of assemblages (whether occurrence‐based or abundance‐based are examined) established in this study echoes published literature highlighting the role of valley‐scale hydrogeomorphology on the variation in fish assemblages in river macrosystems (Boys & Thoms, 2006). However, variation in functional β‐diversity metrics was poorly associated with hydrogeomorphology in our study. Whether this is due to the set of traits used in this analysis or to the relatively low number of taxa in some FPZs is currently unknown. An additional examination of these river networks will be needed to confirm how valley‐scale hydrogeomorphology affects changes in the functional composition of fish assemblages. Accordingly, we believe that our findings of associations between fish assemblage variation and hydrogeomorphic gradients in river networks only partially support our second hypothesis.

We expected to observe differences in the associations between assemblage variation and hydrogeomorphology among the two ecoregions. However, similar patterns (except for the occurrence‐based taxonomic variation as discussed above) were found across the two ecoregions. Certainly, our results account only for valley‐scale hydrogeomorphology and did not include other major drivers of change in fish assemblages like reach‐scale habitat structure, differences in energy fluxes, or food webs. Nevertheless, we can presume that in both ecoregions valley‐scale hydrogeomorphology plays an essential role in structuring fish assemblages at a macrosystem scale. This challenges, therefore, our third hypothesis.

Additionally, we would like to underline that the findings of this study can have substantial implications in informing aquatic biodiversity management and conservation strategies. In the case of Mongolia, the vast majority of rivers are still considered relatively pristine compared to global rivers. However, recent developments in livestock production (Jamsranjav et al., 2018; Maasri & Gelhaus, 2011), extensive recreational fishing (Kaus et al., 2019), increased mining activities (Batsaikhan et al., 2017; Stubblefield et al., 2005), and potential hydroelectric projects threaten the biodiversity and ecological integrity of these river networks. Our results showing variation in fish assemblages across FPZs provide, therefore, a baseline for conservation efforts in these understudied river networks. We confirm that biodiversity patterns are strongly associated with valley‐scale hydrogeomorphology, where the alteration of the latter can result in significant changes in fish assemblages and potentially lead to losses in biodiversity.

## CONFLICT OF INTEREST

The authors declare no conflict of interest.

## AUTHOR CONTRIBUTIONS

**Alain Maasri:** Conceptualization (lead); Data curation (equal); Formal analysis (lead); Funding acquisition (equal); Investigation (equal); Methodology (equal); Project administration (equal); Writing‐original draft (lead). **Mark Pyron:** Conceptualization (equal); Data curation (lead); Funding acquisition (equal); Investigation (lead); Methodology (equal); Project administration (equal); Writing‐original draft (equal). **Emily R. Arsenault:** Conceptualization (supporting); Data curation (equal); Investigation (equal); Methodology (equal); Writing‐original draft (equal). **James H. Thorp:** Conceptualization (equal); Funding acquisition (lead); Methodology (equal); Project administration (equal); Writing‐review & editing (equal). **Bud Mendsaikhan:** Investigation (supporting); Methodology (supporting); Writing‐review & editing (supporting). **Flavia Tromboni:** Investigation (supporting); Writing‐review & editing (supporting). **Mario Minder:** Investigation (supporting); Writing‐review & editing (supporting). **Scott J. Kenner:** Funding acquisition (equal); Investigation (supporting); Project administration (equal); Writing‐review & editing (supporting). **John Costello:** Investigation (supporting); Writing‐review & editing (supporting). **Sudeep Chandra:** Funding acquisition (equal); Investigation (supporting); Project administration (equal); Writing‐review & editing (supporting). **Amarbat Otgonganbat:** Investigation (supporting); Writing‐review & editing (supporting). **Bazartseren Boldgiv:** Funding acquisition (equal); Project administration (equal); Writing‐review & editing (supporting).

## Supporting information

Appendix S1

Appendix S2

## Data Availability

Fish abundance data and sampling coordinates are available on the Dryad Digital Repository https://doi.org/10.5061/dryad.jwstqjq7g.
