# Supplementary material for: Valley‐scale hydrogeomorphology drives river fish assemblage variation in Mongolia
Source: Ecol Evol. 2021 Mar 30;11(11):6527–35. doi: 10.1002/ece3.7505 (PMC8207391; doi:10.1002/ece3.7505)
Supplement: Supplementary file 1 — Appendix S1 [file ECE3-11-6527-s001.docx]

**Valley-scale hydrogeomorphology drives river fish assemblage variation in Mongolia**

Alain Maasri, Mark Pyron, Emily R. Arsenault, James H. Thorp, Bud Mendsaikhan, Flavia Tromboni, Mario Minder, Scott J. Kenner, John Costello, Sudeep Chandra, Amarbat Otgonganbat, Bazartseren Boldgiv

**Appendix S1**: Occurrences, traits, and trait modalities for fish species collected in this study.

**Section S1.1**: Table indicating species occurrences of fishes collected in the forested steppe (FS) and grassland (G) ecoregions of Mongolia by FPZ.

| Species | FS1 | FS3 | FS4 | FS5 | G1 | G2 | G4 | G5 | G6 |
| --- | --- | --- | --- | --- | --- | --- | --- | --- | --- |
| *Barbatula toni* | 1 | 1 | 1 | 1 | 1 |  | 1 | 1 | 1 |
| *Brachymystax lenok* | 1 | 1 | 1 | 1 | 1 |  |  |  | 1 |
| *Carassius gibelio* |  |  |  |  |  | 1 | 1 |  |  |
| *Cobitis melanoleuca* |  |  |  |  | 1 | 1 | 1 | 1 | 1 |
| *Esox lucius* |  |  | 1 | 1 |  |  |  |  |  |
| *Gobio gobio cynocephalus* |  |  |  |  | 1 | 1 | 1 | 1 | 1 |
| *Hemiculter leucisculus warpachovskii* |  |  |  |  |  | 1 |  |  |  |
| *Leuciscus leuciscus* | 1 |  |  | 1 |  |  |  |  |  |
| *Leuciscus waleckii* |  |  |  |  |  | 1 | 1 |  | 1 |
| *Lota lota* |  |  |  |  | 1 |  |  |  | 1 |
| *Misgurnus mohoity* |  |  | 1 | 1 |  | 1 | 1 | 1 |  |
| *Parasilurus asotus* |  |  |  |  |  | 1 | 1 |  |  |
| *Perca fluviatilis* |  |  |  | 1 |  |  |  |  |  |
| *Phoxinus percnurus* |  |  |  |  |  | 1 |  |  |  |
| *Phoxinus phoxinus* | 1 | 1 | 1 | 1 | 1 |  | 1 | 1 | 1 |
| *Pseudaspius leptocephalus* |  |  |  |  |  | 1 |  |  |  |
| *Pseudorasbora parva* |  |  |  |  |  | 1 | 1 |  |  |
| *Rhodeus sericeus* |  |  |  |  |  |  | 1 |  |  |
| *Rhynchocypris lagowskii* |  |  |  |  | 1 | 1 | 1 | 1 | 1 |
| *Thymallus baicalensis* | 1 | 1 | 1 | 1 |  |  |  |  |  |
| *Thymallus grubei* |  |  |  |  | 1 |  |  |  | 1 |

We collected 21 out of 33 fish species predicted to occur in Mongolian FS and G river networks. The twelve species we did not collect are for the FS: Arctic whitefish (*Coregonus pidschian*), Burbot (*Lota lota*), Siberian sturgeon (*Acipenser baerii*), and Taimen (*Hucho taimen*); and for the G: Amur carp (*Cyprinus carpio haematoptrus*), Amur pike (*Esox reichertii*), Amur spiny bitterling (*Acheilognathus asmussi*), Barbel steed (*Hemibarbus labeo*), Blunt snout lenok (*Brachymystax tumensis*), Czekanowskii’s minnow (*Rhynchocypris czekanowskii*), Spotted steed (*Hemibarbus maculatus*), Taimen (*Hucho taimen*), and Topmouth culter (*Culter alburnus*)

**Section S1.2**: Traits and trait modalities used in this manuscript. Trait codes given are used to describe the traits associated with each observed fish species listed in Table S1.3.

| Trait | Modalities | Code |
| --- | --- | --- |
| Maximal total length | <20 cm | ML1 |
|  | 20 to 50 cm | ML2 |
|  | 51 to 100 cm | ML3 |
|  | >100 cm | ML4 |
| Maturity age (years) | <2 years | MA1 |
|  | 2 to 4 years | MA2 |
|  | >4 years | MA3 |
| Longevity (years) | <5 years | Lon1 |
|  | 5 to 10 years | Lon2 |
|  | >10 years | Lon3 |
| Fecundity (number of eggs) | <5k | Fec1 |
|  | 5k to 50k | Fec2 |
|  | >50k | Fec3 |
| Affinity to substrate | Muck/clay/silt substrate | Sub1 |
|  | Sand substrate | Sub2 |
|  | Gravel substrate | Sub3 |
|  | Cobble substrate | Sub4 |
|  | Boulder substrate | Sub5 |
|  | Bedrock substrate | Sub6 |
|  | Aquatic vegetation | Sub7 |
|  | Organic debris or detrital substrate | Sub8 |
|  | Large woody debris | Sub9 |
|  | Pelagic (open water) | Sub10 |
| Affinity to current | Slow current | Cur1 |
|  | Moderate current | Cur2 |
|  | Fast current | Cur3 |
| Feeding guilds | Invertivore | Fee1 |
|  | Piscivore | Fee2 |
|  | Omnivore | Fee3 |
|  | Generalist | Fee4 |
|  | Detritivore | Fee5 |
|  | Algivore | Fee6 |
| Longitudinal distribution | Lowland | Ldis1 |
|  | Upland | Ldis2 |
| Habitat type | Medium to large river | Hab1 |
|  | Stream to small river | Hab2 |
|  | Spring or subterranean water | Hab3 |
|  | Creek | Hab4 |
| Reproductive guild | Non-guarder | Rep1 |
|  | Guarder | Rep2 |
|  | Bearer | Rep3 |

**Section S1.3**: Trait assignments for each fish species collected as part of this study. Trait codes are described in Table S1.2.

|  | *Barbatula toni* | *Brachymystax lenok* | *Carassius gibelio* | *Cobitis melanoleuca* | *Esox lucius* | *Gobio gobio cynocephalus* | *Hemiculter leucisculus warpachovskii* | *Leuciscus leuciscus* | *Leuciscus waleckii* | *Lota lota* | *Misgurnus mohoity* | *Parasilurus asotus* | *Perca fluviatilis* | *Phoxinus percnurus* | *Phoxinus phoxinus* | *Pseudaspius leptocephalus* | *Pseudorasbora parva* | *Rhodeus sericeus* | *Rhynchocypris lagowskii* | *Thymallus baicalensis* | *Thymallus grubei* |
| --- | --- | --- | --- | --- | --- | --- | --- | --- | --- | --- | --- | --- | --- | --- | --- | --- | --- | --- | --- | --- | --- |
| ML1 | 1 |  |  | 1 |  |  |  |  |  |  | 1 |  |  | 1 | 1 |  | 1 | 1 |  |  |  |
| ML2 |  |  | 1 |  |  | 1 | 1 | 1 | 1 |  |  |  | 1 |  |  |  |  |  | 1 |  | 1 |
| ML3 |  | 1 |  |  |  |  |  |  |  |  |  | 1 |  |  |  | 1 |  |  |  | 1 |  |
| ML4 |  |  |  |  | 1 |  |  |  |  | 1 |  |  |  |  |  |  |  |  |  |  |  |
| MA1 |  |  |  |  |  |  |  |  |  |  |  |  |  |  |  |  |  |  |  |  |  |
| MA2 | 1 |  | 1 | 1 | 1 | 1 | 1 | 1 | 1 | 1 | 1 | 1 | 1 | 1 | 1 |  | 1 | 1 | 1 |  | 1 |
| MA3 |  | 1 |  |  |  |  |  |  |  |  |  |  |  |  |  | 1 |  |  |  | 1 |  |
| Lon1 |  |  |  |  |  |  |  |  |  |  |  |  |  |  |  |  |  |  |  |  |  |
| Lon2 |  |  |  |  |  |  |  |  |  |  |  |  |  |  |  |  |  |  |  |  |  |
| Lon3 |  |  |  |  | 1 |  |  |  |  | 1 |  |  |  |  |  |  |  |  |  | 1 |  |
| Fec1 |  |  |  |  |  | 1 |  |  |  |  |  |  |  | 1 | 1 |  | 1 | 1 | 1 |  |  |
| Fec2 |  | 1 | 1 |  |  |  |  | 1 | 1 |  |  |  |  |  |  |  |  |  |  | 1 |  |
| Fec3 |  |  |  |  | 1 |  |  |  |  | 1 |  | 1 | 1 |  |  | 1 |  |  |  |  |  |
| Sub1 |  |  | 1 |  | 1 |  |  |  |  |  |  | 1 |  | 1 |  |  | 1 | 1 |  |  |  |
| Sub2 | 1 |  | 1 | 1 | 1 |  | 1 | 1 | 1 |  | 1 |  | 1 | 1 | 1 | 1 | 1 | 1 | 1 |  |  |
| Sub3 | 1 | 1 | 1 |  | 1 | 1 | 1 | 1 | 1 | 1 |  |  | 1 |  | 1 | 1 |  |  | 1 | 1 | 1 |
| Sub4 |  | 1 | 1 |  | 1 | 1 | 1 |  | 1 | 1 |  |  | 1 |  | 1 | 1 |  |  | 1 | 1 | 1 |
| Sub5 |  |  |  |  | 1 |  | 1 |  | 1 |  |  |  | 1 |  | 1 | 1 |  |  | 1 |  |  |
| Sub6 |  |  |  |  |  |  | 1 |  |  | 1 |  |  | 1 |  | 1 | 1 |  |  |  |  |  |
| Sub7 | 1 |  | 1 |  | 1 |  |  |  | 1 | 1 |  | 1 | 1 | 1 |  |  | 1 |  |  |  |  |
| Sub8 |  |  | 1 |  | 1 |  |  |  |  | 1 |  | 1 |  |  |  |  | 1 |  |  |  |  |
| Sub9 |  |  |  |  |  |  |  |  |  |  |  |  |  |  |  |  |  |  |  |  |  |
| Sub10 |  | 1 | 1 |  |  |  | 1 | 1 | 1 |  |  | 1 | 1 |  |  | 1 | 1 |  | 1 | 1 |  |
| Cur1 |  |  | 1 | 1 | 1 | 1 | 1 | 1 |  | 1 | 1 | 1 | 1 | 1 |  |  | 1 | 1 | 1 | 1 |  |
| Cur2 |  | 1 | 1 |  |  | 1 | 1 | 1 | 1 |  |  |  |  |  | 1 | 1 |  |  |  | 1 | 1 |
| Cur3 | 1 | 1 |  |  |  | 1 | 1 | 1 | 1 |  |  |  |  |  | 1 | 1 |  |  |  |  | 1 |
| Fee1 | 1 | 1 |  | 1 |  | 1 |  |  | 1 |  | 1 | 1 | 1 |  |  |  |  |  |  | 1 | 1 |
| Fee2 |  | 1 |  |  | 1 |  |  |  |  | 1 |  | 1 |  |  |  | 1 |  |  |  |  |  |
| Fee3 |  |  | 1 |  |  |  | 1 | 1 |  |  |  |  |  | 1 | 1 |  | 1 |  | 1 |  |  |
| Fee4 |  |  |  |  |  |  |  |  |  |  |  |  |  |  |  |  |  |  |  |  |  |
| Fee5 |  |  | 1 |  |  |  | 1 |  |  |  |  |  |  |  |  |  |  |  |  |  |  |
| Fee6 |  |  |  |  |  |  |  |  |  |  |  |  |  |  |  |  |  |  |  |  |  |
| Ldis1 | 1 | 1 | 1 | 1 | 1 | 1 | 1 | 1 | 1 | 1 | 1 | 1 | 1 | 1 |  | 1 | 1 | 1 | 1 | 1 | 1 |
| Ldis2 | 1 | 1 | 1 |  | 1 | 1 | 1 | 1 | 1 | 1 |  | 1 | 1 |  | 1 | 1 |  |  | 1 | 1 | 1 |
| Hab1 | 1 | 1 | 1 | 1 | 1 | 1 | 1 | 1 | 1 | 1 | 1 | 1 | 1 | 1 | 1 | 1 | 1 |  | 1 | 1 | 1 |
| Hab2 | 1 | 1 | 1 | 1 |  | 1 | 1 | 1 | 1 |  | 1 | 1 | 1 | 1 | 1 | 1 | 1 | 1 | 1 | 1 | 1 |
| Hab3 |  |  |  |  |  |  |  |  |  |  |  |  |  |  |  |  |  |  |  |  |  |
| Hab4 | 1 | 1 | 1 | 1 |  | 1 | 1 | 1 | 1 |  | 1 | 1 | 1 | 1 | 1 | 1 |  |  | 1 | 1 | 1 |
| Rep1 | 1 | 1 | 1 | 1 | 1 | 1 | 1 | 1 | 1 | 1 | 1 | 1 | 1 | 1 | 1 | 1 |  | 1 | 1 | 1 | 1 |
| Rep2 |  |  |  |  |  |  |  |  |  |  |  |  |  |  |  |  | 1 |  |  |  |  |
| Rep3 |  |  |  |  |  |  |  |  |  |  |  |  |  |  |  |  |  |  |  |  |  |
