# Supplementary material for: Valley‐scale hydrogeomorphology drives river fish assemblage variation in Mongolia
Source: Ecol Evol. 2021 Mar 30;11(11):6527–35. doi: 10.1002/ece3.7505 (PMC8207391; doi:10.1002/ece3.7505)
Supplement: Supplementary file 2 — Appendix S2 [file ECE3-11-6527-s002.docx]

**Valley-scale hydrogeomorphology drives river fish assemblage variation in Mongolia**

Alain Maasri, Mark Pyron, Emily R. Arsenault, James H. Thorp, Bud Mendsaikhan, Flavia Tromboni, Mario Minder, Scott J. Kenner, John Costello, Sudeep Chandra, Amarbat Otgonganbat, Bazartseren Boldgiv

**Appendix 2:** Valley-scale hydrogeomorphic variables and their contribution to the FPZs delineations

**Appendix 2.1:** Boxplots showing the distribution of values of the 10 variables used in the FPZ delineation in forest-steppe (FS) and grassland (G) river networks. Geology is the only categorical variable.

**Appendix 2.2:** Histograms showing the contribution of variables to the first two components of the Principal Component Analysis used in the FPZ delineation. Variables are listed from left to right as the most contributive variable to the least. Contribution values are in relative percentage. Plot FS is for the forest-steppe river network and plot G for the grassland river network.

**Appendix 2.3:** Boxplots showing the distribution of values for each variable characterizing the different FPZs in forest-steppe (FS) and grassland (G) river networks. The numbers and colors of the FPZs correspond to the one used in Figures 1 and 2 in the main manuscript.

**FPZs - Forest-steppe rivers**

FPZs sampled for fish assemblages: F1, F4, F5

**FPZs - Grassland rivers**

FPZs sampled for fish assemblages: G1, G2, G4, G5, G6
